# The role of health policy in the burden of breast cancer in Brazil

**DOI:** 10.1186/s12905-017-0477-9

**Published:** 2017-11-28

**Authors:** Francisco Winter dos Santos Figueiredo, Tábata Cristina do Carmo Almeida, Débora Terra Cardial, Érika da Silva Maciel, Fernando Luiz Affonso Fonseca, Fernando Adami

**Affiliations:** 1 Epidemiology and Data Analysis Laboratory, Faculdade de Medicina do ABC, Santo André, Brazil; 2 Clinical Analysis Laboratoy, Faculdade de Medicina do ABC, Santo André, Brazil; 3 Federal University of Tocantins, Tocantins, Brazil

**Keywords:** Breast cancer, Cost of Health Care, Health Economics, Health Promotion/Disease Prevention, Public Health

## Abstract

**Background:**

Breast cancer affects millions of women worldwide, particularly in Brazil, where public healthcare system is an important model in health organization and the cost of chronic disease has affected the economy in the first decade of the twenty-first century. The aim was to evaluate the role of health policy in the burden of breast cancer in Brazil between 2004 and 2014.

**Methods:**

Secondary analysis was performed in 2017 with Brazilian Health Ministry official data, extracted from the Department of Informatics of the National Health System. Age-standardized mortality and the age-standardized incidence of hospital admission by breast cancer were calculated per 100,000 people. Public healthcare costs were converted to US dollars. Regression analysis was performed to estimate the trend of breast cancer rates and healthcare costs, and principal component analysis was performed to estimate a cost factor. Stata® 11.0 was utilized.

**Results:**

Between 2004 to 2014, the age-standardized rates of breast cancer mortality and the incidence of hospital admission and public healthcare costs increased. There was a positive correlation between breast cancer and healthcare public costs, mainly influenced by governmental strategies.

**Conclusions:**

Governmental strategies are effective against the burden of breast cancer in Brazil.

## Background

In the first decade of the twenty-first century, the Brazilian health system has found difficulties in maintaining stable funding sources for health [1], and the costs of overall public health have been directly affected by chronic diseases [2]; however, the relation with breast cancer is unclear.

Breast cancer continues to be an eminent problem of public health in middle-income countries in which several specific initiatives has been aimed to improve quality and reduce disparities in access of care [3].

In Brazil, where breast cancer is the major cancer in women mainly after 50 years of age [4], disparities in the distribution of wealth affect the overall health of the population [5]; approximately 75% of people have access to healthcare only through public care, and 45.7% of total health expenditures are spent on public health [3].

The incidence of breast cancer has increased with the diagnosis investment, and mortality is associated with management of this disease, but have they influenced the costs of Brazilian public health? In addition, has Brazilian health policy been effective against the burden of breast cancer?

Thus, it is important to describe the trend of mortality, incidence, and the mortality-to-incidence ratio to understand the trend of these epidemiological indicators and their correlation with the healthcare costs of Brazilian public health in this period.

The aim of this study was to evaluate the role of health policy in the burden of breast cancer in Brazil between 2004 and 2014.

## Methods

Secondary analysis was performed in February 2017 with data from the Brazilian Health Ministry. We obtained data extracted from the Mortality Information System (SIM) and Hospital Information System (SIH) from the “*Departamento de Informática do Sistema Único de Saúde*” (DATASUS) website (http://www.datasus.gov.br), to perform the standardization and allow a comparison of rates, available for public access at the DATASUS. The quality of this information system was assessed, in which only 7% of deaths were identified as being from undefined causes [6].

Breast cancer was classified using the 10th revision of the International Classification of Disease – ICD10 [7] – by C50 code. Deaths by breast cancer were extracted from the Mortality Information System (*Sistema de Informações sobre Mortalidade,* SIM), hospital admissions were extracted from the Hospital Information System (*Sistema de Informações Hospitalares,* SIH) and the female population was extracted from the Brazilian Institute of Geography and Statistics (*Instituto Brasileiro de Geografia e Estatística*, IBGE), stratified by year (from 2004 to 2014) and age group.

Age-standardized mortality and the incidence of hospital admissions per 100,000 inhabitants were estimated according to the World Health Organization (WHO) [8]. The mortality-to-incidence ratio (MIR) was calculated as the ratio of deaths to hospital admissions.

The consolidated costs of public health were obtained from the Information System on Public Budget in Health (SIOPS). We extracted the costs of primary care, ambulatory and hospital care, therapeutic and prophylactic care and epidemiologic surveillance, stratified by year and converted into US dollars (quoted at R$ 3.5 each 1,0 R$ according to “Banco Central do Brasil”, accessed in June 2016).

To estimate the trend of breast cancer mortality, incidence, MIR and the consolidated costs of public health in Brazil, we used linear regression. To determine the economic factor, we used principal component analysis with rotation; the Kaiser-Meyer-Olkin test [9] was used to identify the sampling adequacy, and Bartlett’s test was used to assess whether the variables, after principal component analysis, presented variance homogeneity. The significance level was 5%, and Stata® 11.0 (Stata Corp., College Station, EUA) was used to perform the statistical analysis.

## Results

Between 2004 and 2014, 135,432 deaths and 475,339 hospital admissions for breast cancer in Brazilian women occurred, representing costs of US 137.54 million in Brazilian public healthcare.

**Fig. 1:**
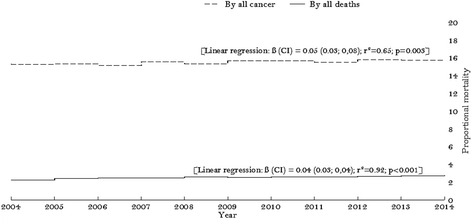
Trend of proportional mortality of breast cancer by all deaths and by all cancer in Brazil between 2004 to 2014

In Brazilian women, in this period, breast cancer represents approximately 15% and 2.7% of deaths by cancer and all deaths, respectively. Annually, this rate is growing, ranging from 15.32 to 15.74 [β = +0.05 (CI 0.03; 0.08), r^2^ = 0.65; *p* = 0.003] deaths by deaths by all cancers from 2004 to 2014, respectively, and from 2.27 to 2.74 [β = +0.04 (CI 0.03; 0.04), r^2^ = 0.92; *p* < 0.001] deaths by all causes of death from 2004 to 2014, respectively (Figure 1).

The age-standardized breast cancer mortality [β = +0.52 (CI 0.49; 0.55), r^2^ = 0.99; p < 0.001] and age-standardized incidence of hospital admission [β = +2.04 (CI 1.49; 2.60), r^2^ = 0.88; *p* < 0.001], respectively, range from 10.73 and 39.42 in 2004 to 15.9 and 58.04 in 2014. The mortality-to-incidence ratio do not range (Table 1).

**Table 1:** Trend of breast cancer rates in Brazilian women between 2004 to 2014

Breast cancer^a^	Year											Linear Regression		
	2004	2005	2006	2007	2008	2009	2010	2011	2012	2013	2014	β (CI 95%)	r^2^	*p*
Age-standardized Mortality^b^ (per 100,000 people)	10.73	11.13	11.87	12.06	12.9	13.04	13.88	14.36	14.78	15.49	15.9	0.52 (0.49;0.55)	0.99	<0.001
Age-standardized Hospital Admission Incidence^b^ (per 100,000 people)	39.42	41.16	41.11	45.51	42.54	44.86	47.82	49.54	54.81	60.61	58.04	2.04 (1.49; 2.60)	0.88	<0.001
Mortality-to-incidence ratio	0.272	0.270	0.288	0.264	0.303	0.290	0.290	0.289	0.269	0.255	0.273	−0.0006 (−0.003; 0.002)	0.02	0.644

Source: Mortality Information System (SIM) and Hospital Information System (SIH/SUS). Data made available by the Department of Informatics of the National Health System (DATASUS—www.datasus.gov.br). Ministry of Health, Brazil

*CI 95%* Confidence Interval of 95%

^a^International classification of diseases, 10th revision. Code C50 [7]

^b^Age-standardized to the WHO standard population [8]

Regarding the trend of costs in public health in Brazil, the costs of hospital and ambulatory care, prophylactic and therapeutic care and epidemiologic surveillance increased. Between 2004 and 2014, the costs of hospital and ambulatory care ranged from US$ 2820.52 million to US$ 12,746.67 [β = +853.1 (CI 687.1; 1019.1), r^2^ = 0.93; *p* < 0.001]; prophylactic and therapeutic care ranged from US$ 231.2 million to US$ 1124.15 [β = +78.4 (CI 54.3; 102.5), r^2^ = 0.85; p < 0.001]; the costs of epidemiologic surveillance ranged from U$ 46.06 million to US$ 142.4 [β = +9.2 (CI 6.3; 12.1), r^2^ = 0.85; *p* < 0.001] (Table 2).

**Table 2:** Trend of Cost with public health in Brazilian public health between 2004 to 2014

Cost*	Year											Linear Regression		
	2004	2005	2006	2007	2008	2009	2010	2011	2012	2013	2014	β (CI 95%)	r^2^	*p*
Primary Care	343.87	411.81	334.8	514.63	560.22	606.96	740.47	764.32	289.73	415.91	464.06	10.5 (−24.8; 45.9)	0.04	0.517
Ambulatory and hospital care	2820.52	3540.82	4455.31	4661.48	5681.43	6760.02	7218.78	7521.46	8408.93	9814.22	12,746.67	853.1 (687.1; 1019.1)	0.93	<0.001
Therapeutic and prophylactic care	231.2	250.59	297.81	407.83	443.49	498.54	446.55	518.17	833.95	832.23	1124.15	78.4 (54.3; 102.5)	0.85	<0.001
Epidemiologic surveillance	46.06	44.91	48.61	49.34	77.29	94.72	94.06	80.53	89.34	128.11	142.4	9.2 (6.3; 12.1)	0.85	<0.001

*CI 95%* Confidence Interval of 95%

*values in US$ million

We found that there was a positive correlation between age-standardized mortality and the health costs related to tertiary care; however, there was not a positive correlation between this index, primary care and the mortality-to-incidence ratio (Table 3).

**Table 3:** Correlation coefficients between breast cancer and Brazilian healthcare costs

Brazilian healthcare costs	Breast cancer^a^					
	Mortality* (per 100,000 people)		Incidence* (per 100,000 people)		Mortality-to-incidence	
	r	p**	r	p**	r	p**
Hospital and ambulatory care	0.966	<0.001	0.917	<0.001	−0.171	0.613
Therapeutic and prophylactic care	0.919	<0.001	0.923	<0.001	−0.320	0.336
Epidemiologic surveillance	0.924	<0.001	0.872	<0.001	−0.131	0.700
Primary care	0.211	0.531	−0.010	0.975	0.540	0.085

Source: Mortality Information System (SIM) and Hospital Information System (SIH/SUS). Data made available by the Department of Informatics of the National Health System (DATASUS—http://www.datasus.gov.br), Ministry of Health, Brazil

r = Pearson’s coefficient

^a^International classification of diseases, 10th revision. Code C50 [7]

*Age-standardized to the WHO standard population [8]

*Pearson’s correlation test

We observed that factor 1 explained 71.9% of the variance of the data, and for this reason, we have considered it in the analysis, representing an economic factor related to tertiary care costs, designated as “healthcare public costs”. The Kaiser-Meyer-Olkin value of greater than 0.5 indicates that this analysis is adequate, and Bartlett’s test for sphericity demonstrates equal variances (*p* < 0.001) (Table 4).

**Table 4:** Principal components analysis of Brazilian healthcare costs and explained variance by factors

Brazilian health costs	Factor 1 Explained variance (71.9%)	Factor 2 Explained Variance (26.1%)	KMO	p*
Hospital and ambulatory care (AHH)	0.990	0.101	0.529	<0.001
Therapeutic and prophylactic care (SPT)	0.980	−0.124		
Epidemiologic surveillance (VE)	0.986	0.155		
Primary care (AB)	0.041	0.998		

*KMO* Kaiser-Meyer-Olkin Measure of Sampling Adequacy

*Bartlett’s test of sphericity

**Fig. 2:**
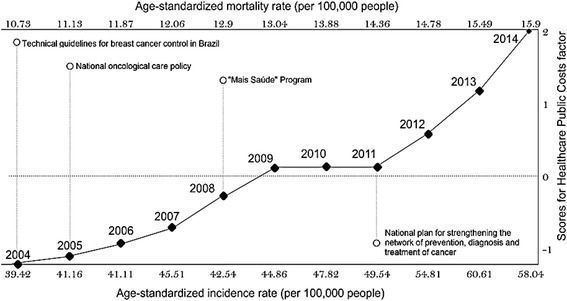
Brazilian government strategies and the burden of breast cancer in Brazil between 2004 to 2014

The economic impact of breast cancer on the found costs of Brazilian public healthcare was that with the increase in age-standardized mortality and age-standardized incidence, the healthcare public cost factor is increased, except between 2009 to 2014 (Figure 2).

## Discussion

We have assessed the correlation between breast cancer and the costs of public healthcare in Brazil between 2004 and 2014, and we have highlighted that the following:

i) The proportional mortality of breast cancer by all cancers and by all deaths has increased;
ii) The age-standardized rates of breast cancer mortality and the incidence of hospital admission have increased;
iii) Public healthcare costs in the period analysed have increased, except for primary care costs;
iv) There is a correlation between the burden of breast cancer and the healthcare costs of Brazilian healthcare.

The limitations in this study included not evaluating breast cancer according to sex, race and economic status. However, this study can be the first research to evaluate the trends in the period characterized by several changes in public health strategies for the management and control of breast cancer in Brazilian women.

The increase in the proportional mortality by all cancers and in the proportional mortality by all deaths can be caused by the delay in breast cancer diagnosis and the increase in Brazilian life expectancy, respectively. This is occurring because Brazil has a large territorial extension affected by regional changes and cultural diversity that can hamper access to healthcare [10].

In addition, it is important to consider that, in Brazil, the distribution of the mortality by breast cancer may vary according to administrative regions. There is a greater occurrence of mortality by breast cancer in the most developed regions (South and Southeast) compared to the more deprived regions (North, Northeast and Central) [11].

In wealthier countries, the early detection of the disease and advances in treatment are the major factors responsible for this difference. Female breast cancer has decreased in the United States since 1989, particularly among younger women, and this decrease has not occurred in Brazil as a whole. [12]

Therefore, the mortality of breast cancer by all cancers and by all deaths has increased, not due only to the high incidence of breast cancer among women, which is first in incidence when excluding non-melanomas, but, rather, due to diagnosis. Hence, there has been a reduction in undefined deaths or wrongly defined deaths.

The age-standardized mortality found in our results is minor compared to the rates observed in Chile, Ecuador, Mexico and Costa Rica [13]. The trends of breast cancer mortality in Brazil were evaluated by Gonzaga et al. [14], who found stability between 1990 to 2011, and by Fonseca et al [15], who found that between 1980 to 2004 there was an increase in breast cancer rates.

However, mammography continues to be a major determinant in preventing mortality by breast cancer. Between 1960 and 1990, eight important randomized clinical trials evaluated the mortality of breast cancer associated with the mammographical screening, with a reduction of 31% in the mortality rate for the group of women subjected to this exam. A meta-analysis of these trials has shown a reduction of 15 to 20% in the relative risk rate of death specifically by breast cancer [16].

In the last 30 years, the Brazilian government has acknowledged the importance of early detection, increasing the number of mammographs available in public services [17]; further, awareness campaigns for the population, such as *“Outubro Rosa”* and the “*mais médicos*” programme, have been crucial in increasing the number of breast cancer diagnoses [18].

According to the INCA - National Cancer Institute of Brazil-, the preoccupation of the Brazilian health system with breast cancer has existed since the 1980s, with specific programmes such as “*Viva Mulher*” programme existing since 2005 and “*Plano de Ações Estratégicas para o Enfrentamento das Doenças Crônicas Não Transmissíveis*” since 2011 [19].

In fact, clinical examination and mammography are recommended for women aged 40 to 69 years and 50 to 60 years, respectively [20], and screening mammography to be conducted every two years is recommended according to the Brazilian Ministry of Health [4].

The problem here is the limitation of access to the follow-up methods defined for breast cancer (mostly mammography). The distribution and access to these prevention methods are not heterogeneously distributed in all of Brazilian territory. Providing geographic access is essential to early diagnosis, which means surgical treatment with the intention to cure; this distribution is responsible for the increase in breast cancer mortality in the country [21].

Unfortunately, a large number of patients receive late diagnosis, in which the only possible treatment is palliative. This difference in the stages of diagnosis can be compared by the percentage of women receiving chemotherapy as treatment against the percentage of those receiving mastectomy. From the economical perspective, it is more advantageous to provide these patients surgery as treatment since neoadjuvant and adjuvant treatment in cancer – particularly breast cancer – is intensively more expensive [22].

However, owing to the growth in higher risk factors, the increase in the incidence found in our study between 2004 and 2014 was already predicted [15]. This increase in incidence could be due to the improvement of sensibility to breast cancer diagnosis [21] or be a consequence of population ageing, the use of hormonal contraceptives methods, exposure to radiation, pesticides and other chemicals and mainly the higher prevalence of risk factors [10, 15].

Lifestyle modifications, such as physical activity, diet, reduction in alcohol consumption, and decrease in body weight and BMI (Body Mass Index), are significant to reduce the risk factors and subsequent costs and breast cancer burden [22].

Excess weight is positively associated with an increase in the circulating level of oestrogen and other circulating hormones and growth factors, which increases the risk of breast cancer and secondary after primary cancer [23]. The reduction in the excessive use of tobacco is a result of public strategies such as applying high rates in the end price of tobacco and prohibiting its use in public places since 1989, when the Brazilian rate of smoking was 32% [20].

Although the trend of abusive alcohol intake is stable in this study, this is a major factor for breast cancer development, in which alcohol intake represents 5% of the etiological factors of this disease [24].

Regarding the mortality-to-incidence ratio, although the index of lethality is stable in the studied period, it still continues to be higher than in high-income countries such as the UK, the USA and European Union and is similar to South America countries. [5]

However, the increase in the costs of hospital and ambulatory care as well as prophylactic and therapeutic care and the stability of basic attention showed that the Brazilian health system has mainly invested in treatment but not in health promotion, in which preventive interventions are the best method of detecting any disease [25].

From the political perspective, the correlation between healthcare costs and breast cancer epidemiology can be explained by governmental strategies for reducing the burden of breast cancer in Brazil.

According to the INCA and performing a cost analysis between years, the increase in investment politics begins in 2004 and 2005, with the implementation of the “*Diretrizes técnicas para controle de cancer de mama no Brasil*” and *“Política Nacional de Atenção Oncológica”,* and continues to increase into 2009, which is one year after of the implementation of the “*Mais Saúde”* programme (2008–2011).

From 2009 to 2011, strategies for the control of health are performed in Brazil, and for this reason, the costs of healthcare associated with breast cancer are established. Only in 2011 do the costs increase due to the implementation of the “*plano nacional de fortalecimento da rede de prevenção, diagnóstico e tratamento do câncer*” [26]. In addition, other aspects, such as the reduction of income inequality, seem to have contributed to changes in the burden of breast cancer in Brazil [27].

## Conclusion

The governmental strategies adopted to improve diagnosis and treatment and to reduce mortality and incidence have been effective against the burden of breast cancer in Brazil.

## Abbreviations

BMI: Body Mass Index
DATASUS: Department of Informatics of the National Health System
IBGE: Brazilian Institute of Geography and Statistics
ICD10: International Classification of Disease
INCA: National Cancer Institute of Brazil
MIR: Mortality-to-Incidence Ratio
SIH: Hospital Information System
SIM: Mortality Information System
SIOPS: Information System on Public Budget in Health
WHO: World Health Organization
